# Development of Database Assisted Structure Identification (DASI) Methods for Nontargeted Metabolomics

**DOI:** 10.3390/metabo6020017

**Published:** 2016-05-31

**Authors:** Lochana C. Menikarachchi, Ritvik Dubey, Dennis W. Hill, Daniel N. Brush, David F. Grant

**Affiliations:** 1 Department of Pharmacy, Faculty of Allied Health Sciences, University of Peradeniya, Peradeniya 20400, Sri Lanka; lochanac@gmail.com; 2 Department of Pharmaceutical Sciences, University of Connecticut, 69 N Eagleville Rd, Storrs, CT 06269, USA; ritvik.k.dubey@gmail.com (R.D.); djhill1967@charter.net (D.W.H.); daniel.brush@uconn.edu (D.N.B.)

**Keywords:** nontargeted metabolomics, mass spectrometry, liquid chromatography, *in silico* structure generation

## Abstract

Metabolite structure identification remains a significant challenge in nontargeted metabolomics research. One commonly used strategy relies on searching biochemical databases using exact mass. However, this approach fails when the database does not contain the unknown metabolite (*i.e.*, for unknown-unknowns). For these cases, constrained structure generation with combinatorial structure generators provides a potential option. Here we evaluated structure generation constraints based on the specification of: (1) substructures required (*i.e.*, seed structures); (2) substructures not allowed; and (3) filters to remove incorrect structures. Our approach (database assisted structure identification, DASI) used predictive models in MolFind to find candidate structures with chemical and physical properties similar to the unknown. These candidates were then used for seed structure generation using eight different structure generation algorithms. One algorithm was able to generate correct seed structures for 21/39 test compounds. Eleven of these seed structures were large enough to constrain the combinatorial structure generator to fewer than 100,000 structures. In 35/39 cases, at least one algorithm was able to generate a correct seed structure. The DASI method has several limitations and will require further experimental validation and optimization. At present, it seems most useful for identifying the structure of unknown-unknowns with molecular weights <200 Da.

## 1. Introduction

Most current metabolomic studies rely on biochemical databases (e.g., Human Metabolite Database (HMDB) [1,2], Kyoto Encyclopedia of Genes and Genomes (KEGG) [3] and Metlin [4]) for structure identification. When high performance liquid chromatography-mass spectrometry (HPLC-MS) is used, the identification strategy typically involves searching these databases with an exact mass and in some cases also using predicted or experimental MS-MS spectra [5,6,7]. Unfortunately, a large percentage of detectable mass spectrometric features observed in biological samples cannot be identified using this approach. This is partially because many of the features being detected are experimental artifacts (adducts, fragments, clusters, *etc.* [8,9]), but is also likely due to the limited number of compounds included in most databases. Searching a large general-purpose chemical database, such as PubChem [10], greatly improves the odds of finding an unknown, provided there is an efficient way to filter out false positives. However, there is still the chance that the correct compound will not be present in PubChem. Additionally, there will always be “unknown” unknowns that cannot be identified by searching databases since there will always be compounds that have never before been identified. Thus, there is a critical need within the metabolomics community to develop automated methods that do not rely on having the correct structure in a database. Combinatorial structure generators [11,12,13,14] provide a means to generate new chemical structures (unknown-unknowns) when a match is not found in an existing database. A combinatorial structure generator enumerates all possible chemical structures for a given elemental formula. However, due to the combinatorial nature of chemical structure generation, the number of output molecules grows exponentially with the number of input atoms. Therefore, generating chemical structures using elemental formulae alone is considered impractical for compounds with more than a few atoms since millions or billions of structures are produced in most cases. To solve this problem, a series of structure generation constraints can be used to limit the combinatorial structure space. For example, a prescribed “seed” structure can be used to limit the structure space to only those containing the “seed” as a substructure. This approach was recently described as a method to generate maximum common substructures (MCSS) that could be used as inputs for constraining combinatorial structure generation [15]. The combinatorial structure space can be narrowed further by eliminating strained ring systems (smaller and larger rings, steric energy index values) and non-endogenous mammalian structures (for biological applications), *i.e.*, by use of a so called “bad list”. This approach was recently used in a semi-automated method along with consensus structure elucidation as an aid in the identification of unknowns in the Critical Assessment of Small Molecule Identification (CASMI) contest [16,17]. Thus, as shown by these studies, if combinatorial structure generators are adequately constrained, they can provide a viable approach for solving the structure identification problem for unknown-unknowns.

The current study focuses on the development of algorithms designed to provide an optimum MCSS or “seed” structure as input for combinatorial structure generators for fully automated *de novo* structure identification using HPLC-MS data. To the best of our knowledge, there is only one previous study [18] that has addressed this problem without using manual interpretation of MS/MS data for confining the structure generation step. In that study, Peironcely *et al.* identified four compounds (out of 30 compounds tested) using HPLC-MS^n^ data and MCSS constrained *de novo* structure generation based on the initial work of Rojas-Cherto *et al.* for seed structure generation [15]. The authors were able to generate “seed” structures for several compounds by matching multi-stage mass spectral trees of unknowns against a database of mass spectral trees. The seed structures were then used as templates to constrain structure generation. The spectral tree database, MetiTree [19] used in that study contained 600 compounds and 900 mass spectral trees. Due to the relatively limited number of spectral trees found in the MetiTree database, the authors were not able to find at least a partial match for 11 compounds. They were able to find partial spectral tree matches (a 10% or better match) for nine compounds, but for five out of these nine compounds, the structure generation could not be sufficiently constrained to yield a manageable number of candidates.

Here we developed several novel “seed” structure generation algorithms. The algorithms identify a consensus seed structure using compounds selected from an initial PubChem database search. The compounds are selected based on having a retention index, Ecom_50_, drift time and collision induced dissociation (CID) spectra (as described [20]) that is similar to the unknown. Thus, these compounds are very close, but not exact matches to the unknown. The proposed database assisted structure identification (DASI) method uses several existing free metabolomics software platforms such as MolFind [20], BioSM [21], MetFrag [22], Parallel Molecular Generator (PMG) [18] and the PubChem database [10]. For this work, we used 40 “putative” unknown-unknowns (*i.e.*, these 40 compounds were removed from the PubChem database prior to searching) ranging in mass from 103 to 608 Da. The implementation details of different seed generation algorithms, combinatorial structure generation, filtering, identification, and limitations of the proposed method are discussed.

## 2. Results

Of the 40 putative unknown unknowns included in this study 39 were usable. In one case, MolFind eliminated all candidates except the unknown (Niacinamide) from the PubChem bin. Table 1 summarizes the performance of the eight different seed generation algorithms. The seed similarity score was calculated as the percent ratio between the number of atoms in the seed structure that exactly matched the target structure (*i.e.*, the unknown). As shown in Table 1, Algorithm-1 (Top MetFrag Fragment from filtered candidates) generated the highest number (24) of correct seed structures. However, only nine of these 24 seed structures lead to fewer than 100,000 combinatorial structures. Of the remaining 15 correct seed structures, there were 10 cases where PMG based structure generation resulted in more than 100,000 structures and five cases where the program failed to generate any structures before it timed out.

The nine targets and PMG seed structures identified with Algorithm-1 are listed in Table 2. The target MIMWs of these nine structures ranged from 103.0633 to 190.0954. The seed to target similarities of the nine structures ranged from 29.2% to 77.7%. Refiltering PMG bins with MolFind eliminated an average of 95% of the incorrect candidates. In 5 out of 9 cases, the correct compound was ranked within the top 10 candidate structures with MetFrag Score ranking. The target monoisotopic molecular weights (MIMWs) of the 15 incorrect seed structures (39 total—24 correct) ranged from 226.1066 to 608.2734. The seed to target similarities of incorrect seed structures ranged from 31.2% to 62.5%.

Algorithm-3–6 generated the second highest (21) number of correct PMG-Seed structures. Of the 21 correct seeds 11 produced fewer than 100,000 PMG structures. In eight cases PMG generated more than 100,000 structures. In the other two cases, PMG failed to generate any structures before it timed out. The putative unknowns identified with Algorithm-3–6 are listed in Table 3. The target MIMWs of correct PMG-seed structures ranged from 117.0790 to 267.0968. All correctly identified putative unknowns except Deoxyguanosine (267.0968) were under 200 Da. The seed to target similarities of the correctly identified putative unknowns ranged from 50.0%–89.5%. In general, different variants of Algorithm-3 resulted in larger seed structures. However, the relatively large seeds generated with Algorithm-3 were not large enough to constrain the structure generation for putative unknowns larger than 200 Da. Refiltering PMG bins with MolFind eliminated on average 86.5% of incorrect candidates. In five out of 11 cases, the correct compound was ranked within the top 10 candidate structures with MetFrag Score ranking. Seed structure data for all eight algorithms are found in the Supplemental Materials.

Even though we used BioSM to eliminate non-endogenous mammalian structures [21], close inspection of the filtered PMG bins that contained more than 250 structures revealed chemical structures with highly strained ring systems. Several options were explored to remove these incorrect chemical structures. In the first attempt, the filtered bins were clustered with 90% Tanimoto structure similarity (using PubChem fingerprints). Then, the average Tanimoto structure similarity between the PMG-Seed and the clusters was used to pick the cluster containing the correct candidate. The Tanimoto clustering with 85%–90% similarity managed to separate out incorrect structures. However, this method failed to pick the cluster containing the correct candidate structure.

As another approach, we used molecular mechanics energies (Table 4) to filter out incorrect structures. Molecular mechanics energies were calculated with force field, MMFF94. The lowest energy conformer generation and MMFF94 based energy minimizations were carried out with ChemAxon’s conformer plugin [23]. An energy cutoff window was established by taking the average and standard deviation of the molecular mechanics energies of the PubChem clusters that lead to the PMG-Seed structures. An approximate energy window (based on the average of relative standard deviations of the other clusters) was established for PubChem clusters with only one structure. PMG candidate compounds whose molecular mechanics energies were outside three times the standard deviation from the average energy were filtered out.

In 4/11 cases, filtering with molecular mechanics energies resulted in improved rankings. In 1/11 cases, the correct candidate was filtered out and in 6/11 cases the ranking was not changed. The latter group of 6 compounds were those that already had good MetFrag scores (average rank = 5). Filtering with molecular mechanics eliminated 58% of the candidates on average. In one case (PubChem chemical ID 11841), filtering with molecular mechanics resulted in 91% reduction of the bin size. The energy based filter improved the average MetFrag Score ranking from 56 to 25.

The DASI method we used relies on having structures in the database that are similar, but not exact matches with the unknown. Thus, it would likely be advantageous to use a large database (such as Pubchem with ~3 × 10^7^ compounds) for this approach. For comparison, the DASI pipeline was repeated using HMDB (~4 × 10^4^ compounds) as the source database. Of the 40 total compounds, 37 were usable as three were no longer included in the latest release of HMDB. For nearly half of these 37 compounds (18 cases), filtering with MolFind resulted in no candidates in the final bin except the putative unknown. In 12 cases, there was one structure other than the putative unknown. The other seven bins had two to five similar structures in the filtered bin. Algorithm-3–6 was able to generate a correct PMG-seed for five bins when using HMDB as the database (as opposed to 21 bins using PubChem). It is important to note that we used 40 HMDB compounds as the test dataset; thus, having some similar compounds (coming from related metabolic pathways) is expected. These results are consistent with our hypothesis that there is an advantage of using a large database (such as PubChem) for the DASI methodology described in this study.

## 3. Discussion

The DASI method is designed to address the common problem encountered in nearly all nontargeted metabolomics studies; how to identify an unknown compound when it is not present in any database. Thus, the approach used here does not require that the chemical database used for the initial search contains the unknown. However, the DASI method does require that the database contains structures that are chemically similar to the unknown (*i.e.*, similar MIMW, RI, Ecom_50_, drift time and predicted CID spectrum); if similar structures are not present, the method will fail. Therefore, as we show using a relatively small database, such as HMDB, the lack of chemically similar structures will limit the utility of the method. Even though very large databases, such as PubChem, are more likely to meet this requirement, there will clearly be some unknown-unknowns where this is not the case.

In this work we make the initial assumption that the elemental formula is known; this assumption is an absolute prerequisite for constraining molecular structure generation algorithms. In a previous study [24], we found that the MolFind approach resulted in the correct formula in the 1st ranked candidate in 98% of 102 tested compounds. In the current study, we found that in 29/39 of the MolFind filtered bins all remaining candidates had the correct molecular formula. Seven bins had one candidate with an incorrect formula and two bins had more than one candidate with an incorrect formula. However, in all of these cases, the most frequently occurring formula in the MolFind filtered bin was the correct one. In only one case was the incorrect formula the most frequent formula in the filtered bin. Even with MolFind, isotope ratios and using instruments with a MIMW accuracy <1 ppm, the probability of selecting an incorrect molecular formula dramatically increases as the MIMW of the unknown increases. If an incorrect elemental formula is used, the method will obviously fail. However, it is important to note that the focus of this work was to systematically address and compare computational issues related to generating seed structures for constraining computational structure generation when the unknown is not present in a database; *i.e.*, for automated *de novo* identification of unknown-unknowns.

Algorithm-3–6 failed to produce a correct PMG-seed structure in 18 of 39 cases, and a large percentage of these were compounds with MIMW > 200 Da. Thus, our results suggest that as the mass of the unknown increases in size (with a corresponding increase in chemical structure diversity), it becomes increasingly difficult to find an identical large seed structure in multiple candidates; *i.e.*, a consensus seed structure becomes less and less likely. At the same time, as unknown unknown compounds become larger, it becomes increasingly more important to constrain structure generation. Given these mutually exclusive limitations, the DASI approach was most useful for compounds with masses below 200 Da. In addition, reasonably similar structures can be lost during the filtering step. For example, in the case of Niacinamide (Predicted RI = 183.7, Predicted Ecom_50_ = 5.49 eV, BioSM Score = 2.0), a similar structure, Picolinamide (Predicted RI = 189.5, Predicted Ecom_50_ = 5.04 eV, BioSM Score = 0.0) was filtered out by the BioSM filter. The only difference between these two structures is the position of the amide group (in Niacinamide, the amide group is meta to the ring nitrogen; in Picolinamide, the amide group is ortho to the ring nitrogen). Limitations like these can be alleviated by improving the predictive models in MolFind. Note that the predictive models we used were given error windows that are currently not achievable. Thus, our results represent a best-case scenario and we assume that our predictive models can be improved as more and more known compounds are added to the modeling process.

Another limitation comes from the lack of unique structural information in the CID spectra of some compounds. For example, for both cytidine and cytidine 5’-monophosphate, their positive ion CID spectra lacked sufficient information to aid in the identification process. A CID spectra search using MassBank [25] revealed that the negative ion CID spectra of these compounds are better suited for identification purposes. The limitations of *in silico* CID prediction algorithms (such as MetFrag used here) also contribute to the overall error. In the cases of cytidine and cytidine 5’-monophosphate, the MetFrag algorithm was able to match only one peak. Improved *in silico* CID fragmentation prediction algorithms would dramatically improve the success rate of DASI.

As already mentioned, there are several limitations of the DASI approach described here. In addition to those listed above, a further limitation of this study is that we were not able to use an independent validation set of compounds. One such option would be to use the set of compounds provided in the CASMI competition mentioned earlier [16]. Unfortunately, the data provided by CASMI are not sufficient to benchmark our method because CASMI was designed for an entirely different purpose. In the CASMI study, the goal was to use MIMW and experimental CID spectra to identify a “known” unknown compound, *i.e.*, one already contained in a database. Our method, on the other hand, relies on the effectiveness of MolFind filtering (using RI, Ecom_50_ and Drift Time models) to identify compounds in PubChem with chemical and physical properties similar to the unknown. However, in our approach the unknown compound is not found in any database; *i.e.*, the unknown is an unknown unknown. Thus, valid benchmarking would require experimental RI, Ecom_50_ and drift times for the benchmark compounds. Alternatively, we could use predicted values for the benchmark compounds and then eliminated them from the database. However, the use of predicted values is essentially what was done in our manuscript. Thus, the benchmark compounds in the CASMI study would serve only to augment the 40 compounds that were chosen in our study.

Perhaps, the biggest limitation of the DASI approach is the inability to verify the validity of the PMG-Seed structure in advance. We attempted to use the cluster averaged MolFind Score as a means to prioritize seed structures, but this approach was largely unsuccessful. Future research will be directed towards addressing this important problem. It is also important to note that the final ranking of the putative unknown can be improved substantially by combining CID matching (MetFrag Score) with other measurements such as RI, Ecom_50_ and drift time (*i.e.*, a MolFind Score). We decided not to report MolFind Score rankings in the current study as calculated RI, Ecom_50_ and drift times tend to inflate the ranking of the putative unknown (in almost all cases, the putative unknown was ranked number 1 with MolFind Score ranking). In practice, with experimental RI, Ecom_50_ and drift time, it is reasonable to expect a good MolFind Score ranking for the correct compound.

## 4. Materials and Methods

The combinatorial structure generation procedure developed in this study involves 4 steps. The steps in order of operation are:

(1) An initial MolFind [20] run using PubChem. Mono isotopic molecular weight (MIMW), retention index (RI), Ecom_50_, drift time and collision induced dissociation spectra (CID) of an unknown are inputted into MolFind’s graphical user interface. MolFind’s built-in QSPR filters and CID spectra predictor MetFrag will provide a ranked list of PubChem candidates that best match the input data in JSON and CSV formats. This step ensures that each of the PubChem candidates selected for subsequent processing (step 2) not only has the correct MIMW, but also has an RI, Ecom_50_, drift time and CID spectrum that closely matches the unknown. Thus, although these compounds are not an exact match to the unknown, they provide a good structural approximation to the unknown. This step also ensures that there are at least some structurally related “hits” in PubChem in order to proceed with seed structure generation.
(2) Output from MolFind (step 1, in JSON format) is fed into the “seed” structure generation program (PMG-Seed); this program will generate one “seed” structure for the combinatorial structure generation program. The seed structure is available in SDF format and represents a consensus structural feature that is shared among the PubChem “hits” identified in step 1 above. For the purposes of this study, during this step the actual unknown and all of its stereoisomers are deleted in order to simulate the unavailability of the unknown in the PubChem database.
(3) Combinatorial structure generation with Parallel Molecular Generator (PMG) [26]; structure generation is controlled by the seed structure and a list of non-endogenous mammalian structures (*i.e.*, structures not allowed in the PMG generated structures). PMG takes the molecular formula, the seed structure (in SDF format) and the list of non-endogenous mammalian structures (in SDF format) as input. PMG’s output is a list of potential unknowns (*i.e.*, candidates) in SDF format all of which have the correct molecular formula, contain the consensus seed structure generated from step 2, and do not contain any non-endogenous mammalian structures.
(4) Finally a second MolFind run is carried out with PMG generated structures from step 3. This step is identical to step 1 above, but instead of filtering PubChem candidates, we are filtering PMG generated candidates using RI, Ecom_50_, drift time and CID spectra matching. Each of these steps is described in more detail below.

### 4.1. Initial MolFind Run

**Figure 1:**
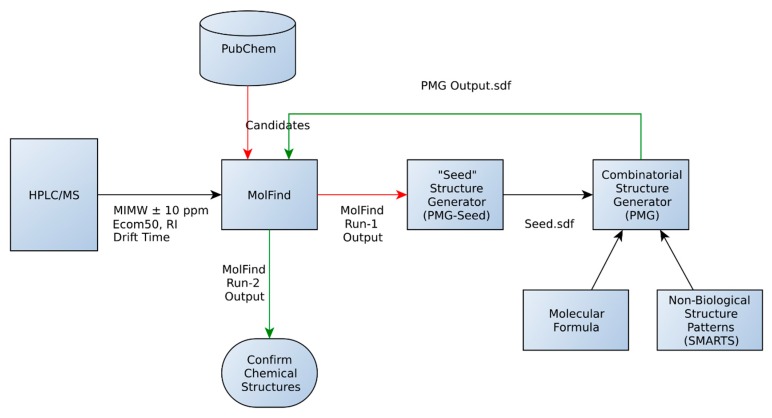
Database assisted structure identification (DASI) flowchart. See text for details.

First, a “bin” of candidate structures matching a MIMW (± mass accuracy of the instrument) is obtained from the PubChem database (Figure 1). The “bin” is then filtered using the computational models (Ecom_50_, RI, Drift Time, BioSM) in MolFind. In a typical MolFind run, experimentally determined MIMW, Ecom_50_, Retention Index (RI) and Drift Time values for an unknown compound are compared with predicted values for candidate compounds from PubChem. Those candidates with predicted values outside the error range of the predictive models are filtered out. However, for the purposes of this study, predicted values (rather than experimental values) for Ecom_50_, RI and Drift Time were used. Predicted values (found in the Supplementary Materials as an Excel file) were used in order to ensure that candidates with chemical and physical properties similar to the unknown (*i.e.*, similar RI, Ecom_50_ and Drift Time) were selected for seed structure generation. Thus, by using predicted values for both the unknown and candidate compounds, we reduced bias associated with using experimental values for the unknown compound and predicted values for candidate compounds. However, each “unknown” was excluded from the candidate list and thus was not used for generating seed structures. The remaining candidates are then fragmented and matched with the experimental CID spectra using the MetFrag algorithm. This results in MolFind Run-1 output (Figure 1), which includes computationally predicted fragments (MetFrag fragments) for filtered candidates. For the purposes of this study, it is assumed that the elemental formula of the unknown is known and the actual unknown or any stereochemically equivalent structure is not present in the PubChem database. Redundant candidates or candidates with an incorrect elemental formula are eliminated.

### 4.2. Seed Structure Generator (PMG-Seed)

Several heuristic algorithms were developed for generating “seed” structures. In the section that follows, each algorithm will be discussed in detail.

(1) Algorithm-1: Top MetFrag Fragment from Filtered Candidates
(2) Algorithm-2: Top MetFrag Fragment from Top PubChem Cluster
(3) Algorithm-3: Intersecting MetFrag Fragments

#### 4.2.1. Algorithm-1: Top MetFrag Fragment from Filtered Candidates

Algorithm-1 is the simplest of the three. This algorithm starts by combining the MetFrag fragments (from different candidates) in the MolFind Run-1 output file into a single set. Then, chemical graphs of these MetFrag fragments are compared to find the unique MetFrag fragments in the set. If a particular fragment occurs multiple times, the number of occurrences and the relative intensity (experimentally measured fragment intensity) of the matched peak are recorded as properties of that unique MetFrag fragment. Finally, MetFrag fragments are sorted by: (1) number of occurrences, and,(2) intensity of the peaks matched; the MetFrag fragment with the highest number of occurrences and greatest intensity is used as the “PMG-Seed” If several fragments have the same number of occurrences, the one with the highest intensity is chosen. If multiple fragments have the same number of occurrences and intensities, the first item in the sorted list is used.

#### 4.2.2. Algorithm-2: Top MetFrag Fragment from Top PubChem Cluster

Algorithm-2 starts by clustering the candidates in the filtered bin. The filtered candidates are clustered based on a 90% Tanimoto structure similarity score. The PubChem structure clustering algorithm was implemented using Chemistry Development Kit (CDK) [27,28] and Hierarchical Agglomerative Clustering (Hac) library from Software and Programmer Efficiency Research Group (SAPE) [29]. These PubChem clusters are then ranked by the cluster averaged MolFind Score. The MolFind Score for a candidate is defined as follows:

$$MolFind\;Score\;=\frac{MetFrag\;Score+\left(1-\frac{\left|\Delta RI\right|}{\left|RI\;Window\right|}\right)+\left(1-\frac{\left|\Delta Ecom_{50}\right|}{\left|Ecom_{50}\;Window\right|}\right)+\left(1-\frac{\left|\Delta Drift\;Time\right|}{\left|Drift\;Time\;Window\right|}\right)}{4}$$

where, ΔRI = Experimental Retention Index − Predicted Retention Index [30,31];
RI Window = Maximum RI value deviation based on model statistics [20] (compounds that are within the experimental RI window are considered potential candidates);
ΔEcom_50_ = Experimental Ecom_50_ (the energy in eV required to fragment 50% of a selected precursor ion) – Predicted Ecom_50_ [31,32,33];
ΔEcom_50_ Window = Maximum Ecom_50_ deviation based on model statistics [20] (compounds that are within Experimental Ecom_50_ ± Ecom_50_ window are considered potential candidates);
ΔDrift Time= Experimental Drift Time – Predicted Drift Time;
Drift Time Window = Maximum Drift Time deviation based on model statistics [20];

Following this, a set of MetFrag fragments is constructed from the members of the top ranked PubChem cluster. The top ranked MetFrag fragment is selected as the “PMG-Seed” structure following the procedure described in Algorithm-1.

#### 4.2.3. Algorithm-3: Intersecting MetFrag Fragments

This algorithm also starts by clustering the filtered candidates by 90% Tanimoto structure similarity. The PubChem clusters are then ranked by the cluster averaged MolFind Score. Then, a maximum common substructure (MCS) is calculated for the top ranked PubChem cluster. Following this, the MCS of the top ranked PubChem cluster is matched with a set of MetFrag fragments. The number of times an atom of the MCS is matched with an atom of a unique MetFrag fragment is recorded. The “PMG-Seed” structure is constructed by deleting a subset of atoms from the MCS (those MCS atoms that had no or fewer matches with MetFrag fragments). Atom removals that lead to 2 or more fragments or disintegration of rings are not allowed. Several variants of Algorithm-3 (Table 5) based on different MetFrag fragment sets and different atom deletion schemes were tested.

**Figure 2:**
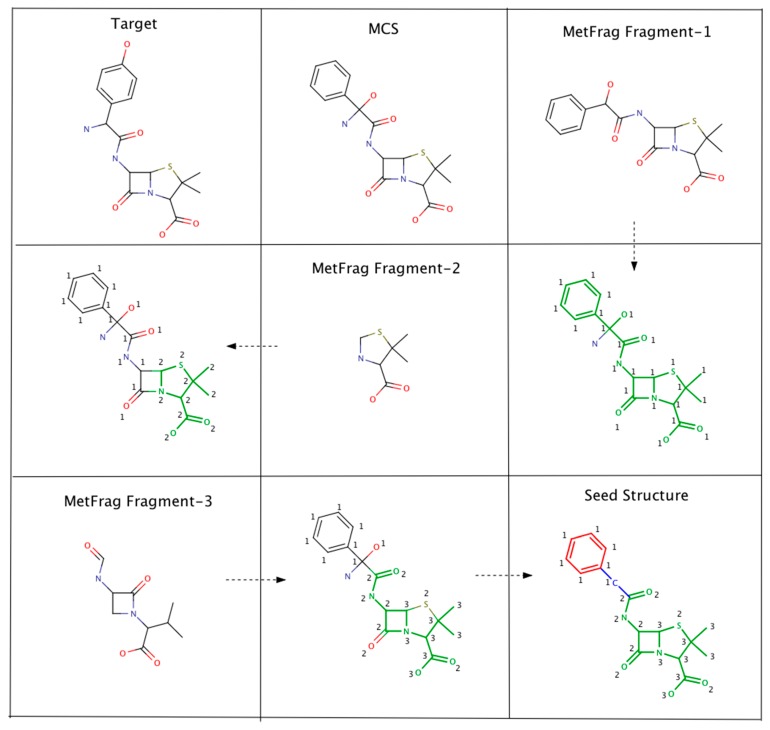
An example illustrating the steps involved in Algorithm 3–3. MCS atoms that matched MetFrag fragments are colored in green. The numbers indicate the number of times a particular MCS atom matched a MetFrag fragment.

As an example, Figure 2 illustrates the steps involved in Algorithm-3–3. The seed structure generation algorithm starts by generating an MCS (for the putative unknown amoxicillin) using PubChem candidates that clustered together and closely matched amoxicillin. Following this, three MetFrag fragments are matched with the MCS while updating atom based matching scores. Finally, the MCS atoms that had fewer than 2 matches are removed. However, the benzene ring (colored in red) and the carbon atom colored in blue are not removed. The red colored atoms (matching score = 1) are kept to avoid the disintegration of the benzene ring. Similarly, the blue colored carbon is retained to prevent the seed structure fragmenting into two pieces.

### 4.3. Combinatorial Structure Generation

The open source combinatorial structure generator, Parallel Molecular Generator (PMG) [26] was used for the structure generation. PMG offers several advantages over other proprietary combinatorial structure generators, such as MolGen [14]. Unlike MolGen, PMG supports multi-threaded architectures allowing a substantial speedup when multiple processor cores are available. Like MolGen, PMG provides a way to generate combinatorial structures around a prescribed seed structure. With a sufficiently large seed substructure, the combinatorial structure space can be narrowed down to a few hundred or thousand structures. Because PMG is written in java and open source, it can be run on any platform and is easily modifiable.

We made two modifications to the original PMG program. In the modified version, the valence of nitrogen was set to 3. In addition, the program was modified to utilize a list of non-endogenous mammalian structures (in SMARTS format). The list of non-endogenous mammalian structures was compiled by collecting strained ring systems and bonding patterns that are not typically found in mammalian systems (Supplemental Table S1). The PMG-Seed structure (the substructure required in all generated structures) and the list of non-endogenous mammalian structures (substructures not allowed in generated structures) were used to constrain structure generation. For the work described here, PMG structure generation was limited to a maximum of 100,000 structures. PMG uses an orderly generation method [34,35] to generate non-duplicate graphs. However, the orderly generation algorithm implemented in PMG does not guarantee non-duplicate graphs when a prescribed seed structure is used. In some cases, generating structures with a prescribed seed structure can lead to millions of duplicate graphs. Removing these structures once they are generated can be prohibitively time consuming (several hours or days to generate the first combinatorial structure). Thus, a timeout (1 h) procedure was used to avoid long running calculations. If PMG failed to output structures after one hour, the structure generation was terminated.

### 4.4. Refiltering PMG Generated Structures

PMG generated structures were refiltered with MolFind’s predictive models and, as described above, the refiltering was done only on PMG generated bins with fewer than 100,000 structures. Refiltering of bins with more than 100,000 candidate structures was considered impractical.

### 4.5. Test Dataset and Calculations

The test data set was comprised of 40 human metabolome database (HMDB) compounds with MIMWs ranging from 103.0633 to 608.2734. CID spectra for each of these 40 test compounds were acquired in positive ion mode. Detailed information on acquisition of CID spectra is presented in the Supplementary Materials. The PubChem bins (MIMW ± 10 ppm) corresponding to putative unknowns were downloaded and filtered using computational models in MolFind. Computationally predicted retention index (RI) [30,31], Ecom_50_ [31,32,33], drift time [20], and BioSM [21] were used in the filtering. The calculations were done with a RI window of ± 40 RI units, Ecom_50_ window of ± 0.5 eV and a drift time window of ± 0.35 ms. The filtered candidates were then fragmented and matched with the experimental CID spectra using the MetFrag algorithm. The output from each MolFind run (in JSON format) was fed into the PMG-Seed program along with an identifier (PubChem ID) and chemical formula of the target. The PMG-Seed structures (in SDF format) were generated with the algorithms described in Section 4.2. The PMG bins that contained fewer than 100,000 structures were refiltered and matched with the experimental CID spectra using MolFind.

## 5. Conclusions

The work presented here describes the use of a database assisted structure identification method for HPLC-MS based metabolomics as a means to identify the structure of an unknown compound when that compound is not found in Metlin, HMDB or any other metabolomics database. In the current study, we evaluated several algorithms for generating “seed” structures (substructures of the target compound). Building structures around a prescribed seed structure and including a large list of not-allowed (*i.e.*, nonbiological) substructures provides constraints to the otherwise intractable combinatorial structure generation problem. One algorithm (Algorithm-1) was able generate correct PMG-Seeds for 24 compounds (out of 39), but only nine of these seeds were large enough to constrain the combinatorial structure generator to fewer than 100,000 structures. Another algorithm (Algorithm-3–6) was able to generate slightly larger correct PMG-Seeds for 21 compounds. Eleven of these relatively larger seeds were able to constrain the combinatorial structure generator to yield a manageable number of candidates. In 35 out of 39 cases, at least one algorithm was able to generate a correct seed structure. Refiltering PMG bins with predictive models in MolFind eliminated 91% of the combinatorial structures on average. Additional filtering of MolFind filtered PMG bins with predicted molecular mechanics energies eliminated 58% of the remaining candidates on average. At the present time, the database assisted structure identification method described in this study is best suited for compounds smaller than 200 Da and should be considered as an aid for structure identification using detailed mass spectral interpretation techniques. The success rate of this method is likely to improve with improved predictive models in MolFind, additional filters, improved CID peak prediction algorithms and more compounds in the PubChem database.

## Figures and Tables

**Table 1:** Number of correct seed structures generated by different seed generation algorithms.

Algorithm	Number of Correct Seed Structures (/39)	Average % Seed Similarity	% Seed Similarity Range
Algorithm-1	24	49.5	31.8–76.9
Algorithm-2	19	49.7	15.7–87.5
Algorithm-3–1	13	71.4	29.4–90.9
Algorithm-3–2	13	71.4	29.4–90.9
Algorithm-3–3	20	66.1	29.4–92.0
Algorithm-3–4	14	63.7	29.4–90.9
Algorithm-3–5	18	69.0	37.5–92.0
Algorithm-3–6	21	66.7	37.5–89.5

**Table 2:** Putative unknowns identified with Algorithm-1.

Target	PMG-Seed	Number of PMG Structures	Number after MolFind	MetFrag Score Rank of the Correct Structure
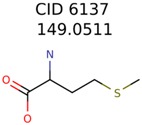	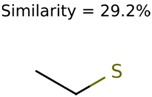	39482	146	11
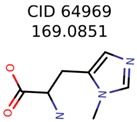	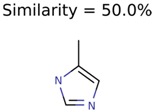	58737	1502	8
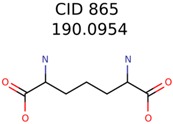	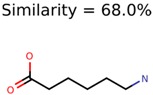	34891	2889	1230
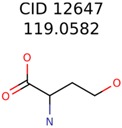	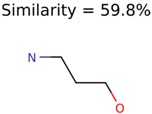	644	40	32
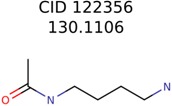	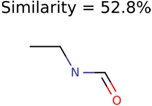	230	33	1
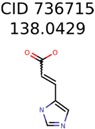	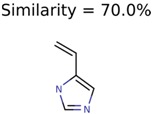	289	2	2
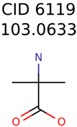	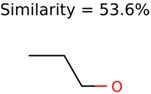	409	15	1
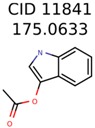	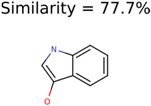	1726	99	3
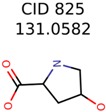	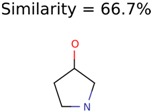	922	30	16

**Table 3:** Putative unknowns identified with Algorithm-3–6.

Target	PMG-Seed	Number of PMG Structures	Number after MolFind	MetFrag Score Rank of the Correct Structure
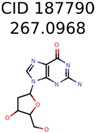	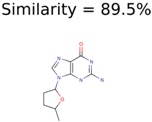	38	2	2
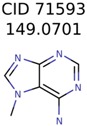	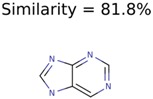	20	3	1
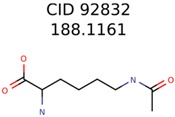	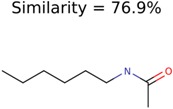	7965	1638	106
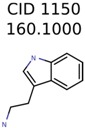	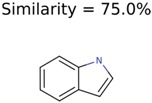	266	8	7
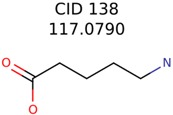	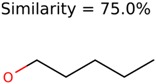	285	48	43
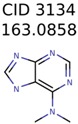	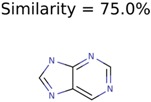	11	4	2
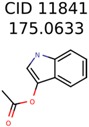	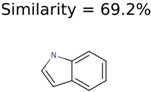	7706	531	24
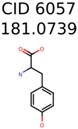	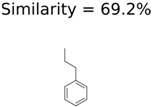	13957	1124	354
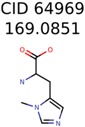	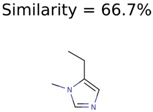	8201	288	5
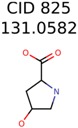	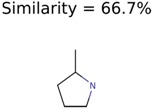	437	22	13
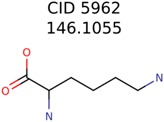	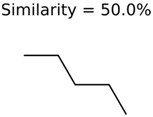	25951	1001	188

**Table 4:** Structure filtering with Molecular Mechanics (MM) energies.

Target PubChem ID	Number of Structures		MetFrag Score Ranking of the Correct Structure	
	Before MM Filter *	After MM Filter	Before MM Filter *	After MM Filter
187790	2	2	2	2
71593	3	3	1	1
92832	1638	367	106	34
1150	8	8	7	7
138	48	26	43	26
3134	4	4	2	2
11841	531	49	24	16
6057	1124	421	354	148
64969	288	163	5	5
825	22	20	13	13
5962	1001	211	188	Filtered Out

* From Table 3.

**Table 5:** Variants of Algorithm-3.

Variant	MetFrag Fragment Set	Atom Deletion Scheme
Algorithm-3–1	Top cluster	Retain MCS atoms with at least 1 match
Algorithm-3–2	All candidates	Retain MCS atoms with at least 1 match
Algorithm-3–3	Top cluster	Retain MCS atoms with at least 2 matches
Algorithm-3–4	All candidates	Retain MCS atoms with at least 2 matches
Algorithm-3–5	Top cluster	Retain MCS atoms with at least average number of atom matches *
Algorithm-3–6	All candidates	Retain MCS atoms with at least average number of atom matches *

* Average number of atom matches is calculated by averaging the number of matches of atoms with at least one match.

## Supplementary Materials

The following are available online at http://www.mdpi.com/2218-1989/6/2/17/s1: (1) A .xlsx file (PMG_Seed_data.xlsx) containing a list of seed structures generated with different algorithms and SMARTS patterns of non-endogenous mammalian substructures, and, (2) a .docx file (Supinfo_Experimental.docx) with detailed information on the acquisition of CID spectra. MolFind, PMG-Seed and modified version of PMG are available to download from http://metabolomics.pharm.uconn.edu/software; Requirements: Java 1.6 or higher, a valid ChemAxon (http://www.chemaxon.com) license.
