# Supplementary material for: Development of Database Assisted Structure Identification (DASI) Methods for Nontargeted Metabolomics
Source: Metabolites. 2016 May 31;6(2):17. doi: 10.3390/metabo6020017 (PMC4931548; doi:10.3390/metabo6020017)
Supplement: Supplementary file 1 [file metabolites-06-00017-s001.zip › metabolites-129772-supplementary/metabolites-129772-supplementary.pdf]

Supplementary Materials: Development of Database Assisted Structure Identification (DASI) Methods for Nontargeted Metabolomics

Lochana C. Menikarachchi, Ritvik Dubey, Dennis W. Hill, Daniel N. Brush and David F. Grant

1. Experimental Section

1.1. Materials

N6-Acetyl-L-lysine, *N*-Acetylputrescine, Aminoadipic acid, 2-Aminoisobutyric acid, 5-Aminopentanoic acid, Amoxicillin, Androstenedione, Apramycin, Brucine, Carnosine, Cyclic AMP, L-Cystine, Cytidine, Cytidine monophosphate, 16-Dehydroprogesterone, Deoxyadenosinemonophosphate, 2′-Deoxyguanosine-5′-monophosphate, Deoxyguanosine, Diaminopimelic acid, 6-Dimethylaminopurine, Flavin Mononucleotide, gamma-Glutamylcysteine, Glycocholic acid, Homocysteine, L-Homoserine, Hydroxyproline, Indoxyl acetate, Inosinic acid, L-Lysine, 7-Methyladenine, 3-Methylhistidine, L-Methionine, Niacinamide, L-Proline, Reserpine, Rolitetracycline, Testosterone, L-Tyrosine, Tryptamine, Urocanic acid were provided by David Wishart (University of Alberta, Edmonton, AB, Canada). HPLC grade acetonitrile (99.9%) was purchased from Fisher Scientific (Pittsburg, PA, USA). Reagent grade water (resistivity 18.2 MΩ-cm) was produced using a Barnstead Nanopure system from Thermo Scientific (Asheville, NC, USA).

1.2. Reagent Preparation

Stock solutions of all the compounds were prepared at 500 μg/mL in water/acetonitrile (1:1). Five mL test samples were prepared individually at appropriate concentrations (*i.e.*, sufficient for adequate sensitivity) in 0.01% (*v*/*v*) trifluoroacetic acid in water/acetonitrile (1:1).

1.3. Instrumentation

The test compounds were analyzed using a Qtof 2 mass spectrometer (Waters Associates, Milford, MA, USA) with a Z-spray electrospray ionization source in positive ion mode. Ion optic parameters were optimized and held constant throughout the study. Nitrogen was used as the desolvation and the collision gas. A head pressure of 12 psi (approximately $6–10 \times 10^{-3}$ mbar collision cell pressure) was used for the collision gas. The desolvation gas flow rate was 250 L/h, whereas the cone gas flow rate was 50 L/h. The source temperature was 120 C and the cone potential was 20 V. The collision cell RF voltage was 180 V. The capillary voltage was 3.2 kV. An acceleration voltage (into the analyzer) of 200 V was used. The MCP detector was set to 2150 V and the TOF flight tube to 9.1 kV. The Qt of 2 uses a quadrupole mass filter in tandem with a hexapole collision cell. A syringe pump (Model # 55-2222, Hamilton Company, Reno, NV, USA) was used to infuse test samples into the electrospray ionization source. MassLynx 4.1 (Waters Associates, Milford, MA, USA) was used to process the spectral data.

1.4. Calculation of SY20 Spectrum

Solutions of test samples were introduced into the electrospray source by flow injection. The $[M + H]^+$ ion of the test compound was analyzed and subjected to collision induced dissociation (CID) analysis. CID spectra were collected at a scan rate of 1 scan/s for 1 min at collisions ranging from 1 to 71 eV at 2 eV intervals. The peak intensities of the resulting CID spectrum at each collision energy were corrected to their theoretical maximum value as outlined by Hill *et al.* [1] and used to calculate the survival yield of the precursor ion (intensity of precursor ion divided by the sum of the precursor and fragment ions intensities). The theoretical SY20 (20% precursor remaining) spectrum was calculated by linear interpolation between the measured CID spectra with the closest SY values less than and greater than SY20. Reserpine and lisinopril were co-analyzed with each test sample.

The precursor and fragment ions of Lisinopril were used to calculate the accurate mass of each ion in the test spectrum and the reserpine control spectrum. The mass of the ions in the control sample was within 10 ppm of their actual mass.

References

1. Hill, D.W.; Baveghems, C.L.; Albaugh, D.R.; Kormos, T.M.; Lai, S.; Ng, H.K.; Grant, D.F. Correlation of $Ecom_{50}$ values between mass spectrometers: effect of collision cell radiofrequency voltage on calculated survival yield. *Rapid Commun. Mass Spectrom.* **2012**, *26*, 2303–2310.
